# Resilience and Associated Factors among Mainland Chinese Women Newly Diagnosed with Breast Cancer

**DOI:** 10.1371/journal.pone.0167976

**Published:** 2016-12-09

**Authors:** Zijing Wu, Ye Liu, Xuelian Li, Xiaohan Li

**Affiliations:** 1 School of Nursing, China Medical University, Shenyang, Liaoning, PR China; 2 Department of Surgical Oncology and Breast Surgery, First Affiliated Hospital of China Medical University, Shenyang, Liaoning, PR China; 3 Department of Epidemiology, School of Public Health, China Medical University, Shenyang, Liaoning, PR China; CNR, ITALY

## Abstract

**Purpose:**

Resilience is the individual’s ability to bounce back from trauma. It has been studied for some time in the U.S., but few studies in China have addressed this important construct. In mainland China, relatively little is known about the resilience of patients in clinical settings, especially among patients with breast cancer. In this study, we aimed to evaluate the level of resilience and identify predictors of resilience among mainland Chinese women newly diagnosed with breast cancer.

**Methods:**

A cross-sectional descriptive study was conducted with 213 mainland Chinese women newly diagnosed with breast cancer between November 2014 and June 2015. Participants were assessed with the Connor-Davidson Resilience Scale (CD-RISC), Social Support Rating Scale (SSRS), Medical Coping Modes Questionnaire (MCMQ, including 3 subscales: confrontation, avoidance, and acceptance-resignation), Herth Hope Index (HHI), and demographic and disease-related information. Descriptive statistics, bivariate analyses and multiple stepwise regression were conducted to explore predictors for resilience.

**Results:**

The average score for CD-RISC was 60.97, ranging from 37 to 69. Resilience was positively associated with educational level, family income, time span after diagnosis, social support, confrontation, avoidance, and hope. However, resilience was negatively associated with age, body mass index (BMI), and acceptance-resignation. Multiple stepwise regression analysis indicated that hope (β = 0.343, P<0.001), educational level of junior college or above (β = 0.272, P<0.001), educational level of high school (β = 0.235, P<0.001), avoidance (β = 0.220, P<0.001), confrontation (β = 0.187, P = 0.001), and age (β = -0.108, P = 0.037) significantly affected resilience and explained 50.1% of the total variance in resilience.

**Conclusions:**

Women with newly diagnosed breast cancer from mainland China demonstrated particularly low resilience level, which was predicted by hope educational level, avoidance, confrontation, and age.

## Introduction

Breast cancer is one of the most prevalent malignancies in women around the world [1]. According to American Cancer Society, approximately 1 in 8 women will develop breast cancer during her lifetime, and more than 2.9 million breast cancer survivors currently reside in the United States [2]. As in most other countries, breast cancer is now the most common cancer in Chinese women; cases in China account for 12.2% of all newly diagnosed breast cancers and 9.6% of all deaths from breast cancer worldwide [3].

Earlier diagnosis of breast cancer in women and the use of systemic adjuvant therapy have increased the likelihood of long term, disease free survival, but the process of diagnosis and treatment can be very taxing for patients. In all phases of the cancer trajectory, from diagnosis and treatment to long-term management, patients may experience physical symptoms, emotional distress, difficulty in interpersonal relationships, and financial strains [4]. Even so, recent studies showed that a substantial proportion of women with breast cancer (43–66%) reported little distress throughout the illness trajectory and appeared to be psychologically resilient, whereas a small subset of women reportedly experienced chronic psychological distress (12–19%) [5–7]. In talking with and listening to the statements of cancer survivors, researchers learned that despite undergoing catastrophic events, many cancer survivors demonstrate remarkable resilience in the face of cancer [8].

Recently, the field of mental health has seen a shift in focus from a deficit-oriented approach to a strength-based approach, which encompasses an interest in the strengths that are associated with healthy adjustment trajectories, such as resilience [9]. Resilience has received considerable attention in oncology nursing [10, 11]. The construct of resilience may account for inter-individual differences in adjusting to cancer. In psychology, resilience is the individual’s ability to bounce back from hardship and trauma [12]. In the face of adversity, resilience can be referred to as protective factors and developmental assets that can help people grow through adversity [13]. Resilience plays an important role in patients’ adjustment to their illness. Patients reporting lower resilience had higher odds of having psychological distress and lower levels of mental health-related quality of life [14]. The recognition of resilience and its usage as a psychological indicator may provide an opportunity for improving psychological outcomes such as anxiety, depression, and quality of life [15].

Resilience has received significant academic interest over the last twenty years [16]. As an emerging field of research, the construct of resilience has been studied in patients with chronic diseases, especially with cancer, in western countries and Hong Kong, China [17–23]. However, in mainland China, resilience research is in a nascent stage, with the first studies in this area published within the last decade, and few studies conducted in clinical settings [24]. Relatively little is known about resilience in mainland Chinese women with breast cancer.

Existing studies have identified a number of factors that contribute to resilience at the individual (e.g., coping strategies, personality, positive emotions, and illness-related factors) and interpersonal (e.g., social support) levels [7, 17, 25–27]. According to the resilience perspective, these variables may function as protective or risk factors in individual’s capacity to deal with adversity [23, 27]. It is important to identify main variables associated with resilience of individual, which may help inform the development of interventions aimed at fostering resilience.

According to the Adolescent Resilience Model (ARM), Haase [23] acknowledged that intervention factors, such as social support, hope and positive coping, can contribute to developing resilience and be manipulated to enhance resilience. The ARM is developed in the population of adolescents with cancer. However, there were very few studies exploring the effects of social support, coping strategy and hope on resilience in adult survivors, especially among mainland Chinese women with breast cancer [10, 21]. Thus, this cross-sectional descriptive study was designed to (1) evaluate the level of resilience among mainland Chinese women with breast cancer; (2) identify factors associated with resilience in this sample of Chinese women; and (3) lay the groundwork for larger, more systematic studies of resilience among Chinese women with breast cancer.

## Methods

### Participants

The cross-sectional study was conducted in the Department of Breast Surgery at the First Hospital of China Medical University between November 2014 and June 2015. The Department of Breast Surgery is one important provider of breast cancer services to people in the northeastern China. Women newly diagnosed with breast cancer, who were consecutively hospitalized for their scheduled treatment postoperatively, were recruited to participate in the study. Subjects were eligible for study participation if they were 18 years old or older, underwent breast surgery, had histologically proven breast cancer, were aware of their own cancer diagnosis, were able to communicate in Chinese language, and had sufficient cognitive ability to participate in the study. Exclusion criteria were patients who had a history of breast cancer recurrence; and patients who had significant organ dysfunction.

All eligible participants provided written informed consent before completing a structured questionnaire. Clinical data were collected from electronic medical records. Initially, a total of 224 patients were recruited, out of which 220 completed and submitted the questionnaire. 7 patients were excluded from analysis because of incomplete data. Final analysis was based on the remaining 213 questionnaires. The study was approved by the Ethics Committee of China Medical University.

### Instruments

#### Connor-Davidson Resilience Scale (CD-RISC)

Psychological resilience was measured with the 25-item Chinese version of the Connor-Davidson Resilience Scale (CD-RISC) [28, 29]. It was translated into Chinese by Yu and Zhang and has been validated among Chinese community residents, which consists of 3 factors: tenacity, strength and optimism [29]. Items (e.g., “Able to adapt to change”; “Tend to bounce back after illness or hardship”) are rated on a 5-point Likert scale, ranging from 0 (“not true at all”) to 4 (“true nearly all the time”). According to the CD-RISC manual, the total score of the entire scale is an indicator of the level of individual resilience. The possible scores of the 25-item scale range from 0 to 100, with higher scores reflecting higher resilience. The Cronbach’s α reliability of the CD-RISC in this study was 0.906.

#### Social Support Rating Scale

Social support was measured with Chinese Social Support Rating Scale (SSRS), which has been used in Chinese populations and proved to have good validity and reliability [30]. The 10-item scale contains 3 subscales: objective support, subjective support and support utilization. The total score ranging from 0 to 66 is used as a measure of current total social support status of the subject, with higher scores indicating better support. The Cronbach’s α reliability for this scale in the study was 0.836.

#### Medical Coping Modes Questionnaire

Coping strategy was measured with the Chinese version of the Medical Coping Modes Questionnaire (MCMQ), which has been widely used to measure patients’ coping strategies [31]. It is a 20-item questionnaire designed to assess 3 types of illness-related coping strategies: confrontation, avoidance, and acceptance-resignation. The question provides 4 responses, ranging from 1(never) to 4 (very often). 8 of the 20 items are reverse-scored. For each of the 3 ways of coping, high scores indicate that the participants often use the behaviors described by that specific coping scale when dealing with medical events. The Cronbach’s α values for the confrontation, avoidance, and acceptance-resignation subscales in this study were 0.626, 0.716, and 0.698, respectively.

#### Herth Hope Index

Trait hope was measured with Chinese version of Herth Hope Index (HHI) [32, 33]. The 12-item scale has 3 subscales: temporality and future, positive readiness and expectancy, and interconnectedness. Each item is rated from 1 (strongly disagree) to 4 (strong agree) and total score ranges from 12 to 48, with higher score reflecting greater hope. The Cronbach’s α value for HHI in the study was 0.858.

#### Demographic and Disease-Related Information

Demographic and clinical variables were collected from participants and hospital charts. The demographic variables of patients included age, body mass index (BMI), marital status, educational level, monthly family income, religion, and insurance status. Disease-Related Information included cancer stage, surgery type, family history of breast cancer, and time span after diagnosis.

### Data analyses

Statistical analyses were conducted with the Statistical Package for the Social Science (SPSS, version 20.0). Significance for all statistical tests was set at 0.05 or less (2-tailed). Descriptive statistics were used to assess sample characteristics and psychological variables. Normality and homogeneity of variances were first tested for each variable. Independent-sample t test and 1-way analysis of variance were conducted to compare the means of resilience for categorical variables. Bivariate analyses were performed between demographic variables, clinical variables, social support, confrontation, avoidance, acceptance-resignation, hope, and resilience to observe changes in regression coefficients when variables were examined by multivariable analysis. Multiple stepwise regression analysis was conducted to identify the predictors of resilience. All significant explanatory variables were entered in the regression analysis at P<0.05 and removed from the model at P>0.10. Collinearity between independent variables was tested based on variance inflation factors and tolerances.

## Results

### Descriptive statistics

Demographic and clinical characteristics of participants were shown in Table 1. The participants (n = 213) ranged in age from 26–67 years (Mean±SD: 47.30±7.87). The median age was 47 years. Approximately 92.5% of the participants were married or living with a partner, and 45.07% received middle school or below. In relation to clinical variables, the mean time span after diagnosis was 85 days (range: 6–483 days). Minorities of women subjects (11.27%) were diagnosed at cancer stageⅢ and none participants were diagnosed with metastasis.

**Table 1:** Demographic and clinical characteristics of participants (n = 213).

Variables		N	%
Age (years)	≤44	76	35.7
	>44	137	64.3
BMI	<24	113	53.1
	≥24	100	46.9
Marital status	With a partner	197	92.49
	Without partner	16	7.51
Educational level	Middle school or below	96	45.07
	High school	46	21.6
	Junior college or above	71	33.33
Monthly family income (RMB)	≤3000	56	26.3
	3001~6000	101	47.4
	>6000	56	26.3
Family history of breast cancer	Yes	18	8.5
	No	195	91.5
Religion	Yes	35	16.4
	No	178	83.6
Stage of cancer	Ⅰor 0	50	23.48
	Ⅱ	139	65.25
	Ⅲ	24	11.27
Surgery type	Mastectomy	196	92
	Conservative surgery	17	8
Time span after diagnosis (days)	≤60	86	40.4
	60~120	84	39.2
	>120	43	20.2

Table 2 provided the level of resilience, social support, coping strategies, and hope among participants. The mean score for resilience of breast cancer patients was 60.97±12.30, ranging from 37 to 96. The mean values were 43.61±6.24 for social support, 18.66±3.48 for confrontation, 18.31±2.45 for avoidance, 7.63±2.19 for acceptance-resignation, and 37.02±3.65 for hope.

**Table 2:** Descriptive statistics for resilience, social support, coping strategies, and hope (n = 213).

Variables	Mean	SD	Range
CD-RISC	60.97	12.30	37–96
SSRS	43.61	6.24	23–59
MCMQ-Confrontation	18.6573	3.48	10–28
MCMQ-Avoidance	18.3099	2.45	10–25
MCMQ-Acceptance-resignation	7.6338	2.19	5–14
HHI	37.02	3.65	27–48

CD-RISC = Connor-Davidson Resilience Scale; SSRS = Social Support Rating Scale; MCMQ = Medical Coping Modes Questionnaire; HHI = Herth Hope Index.

### Effects of demographic and clinical variables on resilience

Table 3 showed resilience scores according to demographic and clinical characteristics. Age, BMI, education level, monthly family income, and surgery type showed statistically significant differences in the level of resilience (Table 3). Participants who were 44 years old or younger reported a higher level of resilience (65.29±11.97) than those older than 44 years (58.58±11.86). Participants whose BMI was less than 24 had a higher level of resilience (62.72±12.40) than the participants whose BMI was greater than or equal to 24 (59.00±11.94). The mean resilience score for patients with education level of middle school or below was 54.93 (SD, 9.42), which was lower than the score for patients with education level of high school (64.15±11.45) or junior college or above (67.08±12.60). The higher monthly family income participants had, the higher level of resilience they reported. Participants receiving conservative surgery had higher scores of resilience (67.12±11.41) than those receiving mastectomy (60.44±12.26).

**Table 3:** Mean scores of resilience according to demographic and clinical characteristics (n = 213).

Variables		CD-RISC Score		
		Mean±SD	t^a^/ F^b^	*p*^*c*^
Total		60.97 ±12.30		
Age (years)	≤44	65.29±11.97	3.945	0.000
	>44	58.58±11.86		
BMI	<24	62.72±12.40	2.222	0.027
	≥24	59.00±11.94		
Marital status	With a partner	61.02±12.21	0.180	0.857
	Without partner	60.44±13.79		
Educational level	Middle school or below	54.93±9.42	27.345	0.000
	High school	64.15±11.45		
	Junior college or above	67.08±12.60		
Monthly family income (RMB)	≤3000	57.34(12.83)	3.393	0.035
	3001~6000	62.20(11.54)		
	>6000	62.40(12.57)		
Family history of breast cancer	Yes	65.44±12.78	1.619	0.107
	No	60.56±12.20		
Religion	Yes	61.83±11.99	0.450	0.653
	No	60.80±12.38		
Stage of cancer	Ⅰor 0	62.88±12.73	0.796	0.453
	Ⅱ	60.33±12.31		
	Ⅲ	60.72±11.38		
Surgery type	Mastectomy	60.44±12.26	-2.166	0.031
	Conservative surgery	67.12±11.41		
Time span after diagnosis (days)	≤60	59.71±11.46	1.520	0.221
	60~120	60.87±13.41		
	>120	63.70±11.45		

^a^ Two-Sample T-Test was used to compare means of two group

^b^ One-way analysis of variance was used to compare means of three or more groups

^c^ P value

### Associations between predictors and resilience

Bivariate analyses were conducted to examine the associations between predictors and resilience, consisting of demographic variables, clinical variables, social support, confrontation, avoidance, acceptance-resignation, and hope. For the categorical variables, we created dummy variables using educational level of middle school or below, monthly family income≤3000, and cancer stage Ⅰ or 0 as the reference group respectively. As shown in Table 4, resilience was positively associated with educational level, family income, time span after diagnosis, social support, confrontation, avoidance, and hope. However, resilience was negatively associated with age, BMI, and acceptance-resignation.

**Table 4:** Associations between predictors and resilience (n = 213).

Variables	coefficients	95% CI	p
Age	-0.427	-0.631, -0.223	0.000
BMI	-0.584	-1.122,-0.047	0.033
Marital status	-0.578	-6.895, 5.739	0.857
Education-High school	9.225	5.334,13.116	0.000
Education-Junior college or above	12.157	8.761,15.554	0.000
Monthly family income 3001~6000	4.859	0.864,8.853	0.017
Monthly family income >6000	5.054	0.523,9.585	0.029
Family history of breast cancer	4.885	-1.064,10.835	0.107
Religion	1.025	-3.466,5.517	0.653
Stage of cancerⅡ	-2.554	-6.560,1.452	0.210
Stage of cancer Ⅲ	-2.160	-8.105,3,785	0.475
Surgery type	6.679	0.602,12.756	0.031
Time span after diagnosis (days)	0.026	0.001,0.051	0.041
Social support	0.398	0.136,0.660	0.003
Confrontation	1.588	1.159,2.017	0.000
Avoidance	1.763	1.127,2.400	0.000
Acceptance-resignation	-1.207	-1.951,-0.463	0.002
Hope	1.928	1.552,2.304	0.000

CI, confidence interval; p, P value.

### Predictors of resilience

Multiple stepwise regression analysis was conducted to identify the predictors of resilience. Variables, which were significantly associated with resilience and reported in the prior studies, were included in the multiple regression analysis, consisting of demographic variables, clinical variables, social support, confrontation, avoidance, acceptance-resignation, and hope. Finally, as shown in Table 5, the variables included into regression model were hope (β = 0.343, P<0.001), educational level of junior college or above (β = 0.272, P<0.001), educational level of high school (β = 0.235, P<0.001), avoidance (β = 0.220, P<0.001), confrontation (β = 0.187, P = 0.001), and age (β = -0.108, P = 0.037). These 5 variables explained 50.1% of the total variance in the participants’ resilience.

**Table 5:** Multiple regression analysis of predictors of resilience (n = 213).

Model	Unstandardized coefficients	Standard error	Standardized coefficients	t^a^	*p*^*b*^
Constant	-10.290	9.024	-	-1.140	.256
Hope	1.158	0.187	0.343	6.172	.000
Junior college or above	7.080	1.454	0.272	4.868	.000
High school	7.015	1.588	0.235	4.418	.000
Avoidance	1.102	0.252	0.220	4.376	.000
Confrontation	0.662	0.196	0.187	3.373	.001
Age	-0.169	0.080	-0.108	-2.105	.037

^a^ the significance test of individual regression coefficient

^b^ P value

R^2^ = 0.515; Adjusted R^2^ = 0.501

## Discussion

Most breast cancer patients receive medical treatment after surgery in an attempt to reduce the risk for cancer recurrence and death [34]. The intense and cyclic chemotherapy requires repeated hospitalization, and can induce many side-effects, such as hair loss, nausea, vomiting and fatigue [35]. The effects of illness and treatment-induced side effects can subject patients to psychological distress [35, 36]. Studies have found cancer patients can benefit from positive psychological-based interventions [37]. Therefore, the construct of resilience as a potential protective factor needs to be considered. Promoting resilience mechanisms may encourage better adaptation and other positive psychological outcome during and after treatment [21].

### Resilience level

The present cross-sectional study evaluated the level of resilience in a sample of 213 mainland Chinese women with breast cancer. The patients investigated in this study reported lower scores of resilience (60.97±12.30) as compared with a normative sample from China(65.4±13.9) [29] and US (80.4±12.8) [28]. Loprinzi et al. [19] reported the score of resilience (73.6±10.1) among American breast cancer survivors, measured by the CD-RISC, which is higher than the degree of resilience in the present study. The inconsistency between these two studies might be due to differences in sample characteristics, such as age and time span after diagnosis. Furthermore, culture may also play a role in resilience differences between Chinese and American populations. Ethnocultural women’s breast cancer experiences were shaped by the social and personal context in which they lived [38]. Confucianism, Taoism and Buddhism constitute the essence of the traditional Chinese culture, which is different from American culture. Therefore, Chinese women differ from western women in preserving psychological integrity when facing adverse event. Further analysis of each item, the item of ‘sometimes fate or God can help’ got the lowest score. It is possible that Chinese women might not have religious beliefs or might not have realized that praying could help them to adapt to breast cancer [39]. This study just used quantitative method to measure individuals’ resilience level, but little is known of the meaning of resilience to Chinese breast cancer patients. Therefore, future qualitative research is needed to explore the meaning of resilience for Chinese women with breast cancer.

### Factors associated with resilience

Multiple regression analysis demonstrated that, in combination, these 5 factors (hope, educational level, avoidance, confrontation, and age) explained 50.1% of the variance in resilience of patients with breast cancer, with self-reported hope making the largest predictive contribution. These findings have not been reported previously in mainland Chinese women with breast cancer and provide useful information for clinical practice and further research.

In this study we found hope was the most significant predictor (β = 0.343, P<0.001) for resilience. Our result indicated positive correlation of resilience with hope, and it is in accordance with previous study findings [17, 40]. A longitudinal study conducted by Ho et al. [17] found hopefulness may predict resilience after hereditary colorectal cancer genetic testing in Hong Kong Chinese. Solano [41] and Haase [40] respectively carried out a cross-sectional study and found positive correlation of resilience with hope among metastatic colorectal cancer patients and adolescent/young adults with cancer. Hope is an inner power energized in the face of adversity, which could enable patients to establish positive and realistic goals, and mobilize resources to positively manage the physical and psychological challenges. Hope can be considered as a protective factor that can buffer the adverse effects of having cancer.

Educational level was an independent predictor (β = 0.286, P<0.001) of resilience in this study. It is consistent with the evidence of a previous study [15], which also found educational level was another direct predictor of resilience. Patients with higher education level might have more access to information about breast cancer by various channels, such as communication with other patients or medical staff and searching the internet. Thus, they have a better understanding of the disease, and gain more feeling of control during the course of treatment. In addition, patients with higher education level might have higher income, which can help them better deal with the problems caused by the disease and treatment. Although income was not included in the regression model, it is very likely that some of the effects of income were also captured in the measure of education.

Avoidance and confrontation were another 2 predictors of resilience, which are subtypes of coping strategies. Of the 2 variables, avoidance coping provided the greater predictive contribution (β = 0.220, P<0.001) to resilience for participants. Confrontation coping was positively correlated with resilience and was a statistically significant predictor (β = 0.187, P = 0.001) of resilience. Coping strategies are often operationalized differently across studies, which prevents a detailed comparison of study findings. Haase [23] pointed out positive coping strategies (including confrontive, optimistic, and supportant coping strategies) were linked to greater resilience and defensive coping strategies (including evasive, fatalistic and emotive) negatively affected resilience. Llewellyn [25] found use of emotional support and active coping strategies were predictive of resilience. Wu [26] reported that defensive coping (β = −0.165, P<0.01) and cognitive coping (β = 0.745, P<0.01) were also statistically significant predictors of resilience. The present result of positive relationship between avoidance coping and resilience is inconsistent with the above study findings [23, 26]. It may be that avoidance coping can make patients focus attention away from negative life events and severity of negative events. Thus, it may decrease the psychological stress and make patients gain a positive outcome. Avoidance coping is similar to defensive coping mechanisms, which may be beneficial for uncontrolled stressors, such as cancer diagnosis. In this study, patients with breast cancer were usually reluctant to talk about their disease and sufferings with others. The phenomenon can be explained by traditional Chinese culture. In China, women are expected to be ‘a good wife and loving mother’ whose role is to maintain harmony within and outside the family [42]. Thus, they make efforts to lessen the troubles and dissonant relations promoted by cancer to alleviate others’ burdens [39, 43]. The present result of positive correlation between confrontation coping and resilience is similar to the above study findings [23, 25, 26]. Facing the reality of disease creates an opportunity to view their current situations from a different perspective and puts a stop to the downward spiral of negativity and self-harm [39, 44]. Positive coping strategies including confrontation and problem-solving can help patients adjust well. It is advantageous for patients to develop confrontation coping and other positive coping strategies to flexibly manage stressors and thus acquire a positive outcome, such as resilience. Although avoidance and confrontation make different contributions to resilience, there appears that coping strategy is important to facilitate resilience development. Further qualitative research should be conducted to examine the mechanisms of coping strategies contributing to or inhibiting resilience under Chinese culture.

Age provided a limited predictive contribution. The negative relationship between age and resilience was modest (β = −0.108, P = 0.036), suggesting that younger patients reported greater resilience. It is inconsistent with previous reports regarding the association between age and resilience [14, 45]. Rosenberg [14] found age was positively associated with resilience and Manne [45] reported there was no difference in resilience among cancer patients based on age. There is a paucity of research examining the association between age and resilience among adults with cancer. Whether younger patients consistently report greater resilience need to be confirmed in additional research in this population.

Furthermore, time span after diagnosis was not predictive of resilience for participants. There is no agreement with the effect of time span since diagnosis on resilience. Manne [45] found women newly diagnosed with gynecological cancers who experienced a longer period of time from diagnosis reported less resilience. However, Schumacher [46] found that the group of patients 3–4 years after alloSCT reported a lower degree of resilience than the group of patients 1–2 years or 5 and more years after alloSCT. For patients with breast cancer, the effect of changes over time after diagnosis on resilience should be examined in prospective studies.

Recently, some literatures suggested that psychological care should be integrated into the routine cancer care of patients [47, 48]. A low degree of resilience of women patients with breast cancer could be an indicator for necessary psychological intervention. This study demonstrated that variables including hope, educational level, avoidance, confrontation, and age were predictors for the level of resilience among mainland Chinese women with breast cancer, and provided important implications for healthcare professionals. It is very important for healthcare providers to recognize that hope plays a significant role in the adjustment to cancer. It may be appropriate to encourage patients to direct hope and expectation towards attainable goals that are meaningful for the individual patient [49, 50]. Educational level was a significant predictor of resilience, which implies that medical personnel should provide enough information related to the disease and establish various channels of communication between patients with cancer and medical personnel and among patients with similar situations. Avoidance and confrontation were predictors for resilience, which indicates the vital role of coping strategies in adjustment to cancer. Age, as another predictor of resilience, is not modifiable through intervention. However, medical staff can recognize the risk for lower resilience in older patients and provide resources to help them. Therefore, this study has provided some preliminary evidence that women with newly diagnosed breast cancer in this convenience sample from mainland China seem to have particularly low resilience scores, and the inconsistent findings with previous reports may reflect cultural patterns that are different from women in the United States. Future qualitative and quantitative research is warranted to thoroughly explore resilience in women with newly diagnosed breast cancer from mainland China.

### Limitation

The study has several limitations that require consideration when interpreting the findings. First, convenience sampling in one setting limited the generalizability of the findings to breast cancer patients. Second, this study was based on cross-sectional design, which limits the development of the causal relationship between resilience and other variables in breast cancer patients. Further prospective and longitudinal studies are necessary to validate the current findings. Third, this study just explored the influence of individual-level factors on resilience. Future studies can be conducted to investigate the effect of both individual- and community-level factors on resilience for patients in clinical settings. Forth, this study was a quantative research, which can provide limited information about resilience, and further qualitative research is necessary to explore the meaning of resilience and identify contributors and inhibitors of resilience among Chinese women with breast cancer from a culturally specific perspective. Finally, we conducted exploratory analysis, which may increase the risk of type error. The current findings should be interpreted with caution since we could not fully consider interactions or additive effects among the various covariates. Therefore, the study results should be considered with caution, and further investigation combined with diverse methods is necessary to explore and understand resilience thoroughly among Chinese women with breast cancer.

## Supporting Information

S1 Data The S1 Data includes the data underlying our findings in this study. (XLS)
